# Incidence of Coronavirus Disease 2019 (COVID-19) among healthcare workers during the first and second wave in the Democratic Republic of the Congo: a descriptive study

**DOI:** 10.1186/s12879-023-08494-4

**Published:** 2023-08-08

**Authors:** Jean Paul Muambangu Milambo, James Ndirangu, Samuel Mangala, Hannah Simba, Landry Kabego

**Affiliations:** 1 Division of Public Health, University of Free State, Bloenfontein, South Africa; 2 Department of Health and Prevention, Kinshasa, Democratic Republic of the Congo; 3 Faculty of Medicine and Health Sciences, Department of Global Health, Stellenbosch University, Cape Town, South Africa; 4 Health Emergencies Programme, WHO, African Region, Democratic Republic of the Congo

**Keywords:** COVID-19, Healthcare workers, Infection Prevention and Control, The Democratic Republic of the Congo

## Abstract

**Background:**

Healthcare workers (HCWs) are at the frontline of response to the COVID-19 pandemic. Protecting HCWs is of paramount importance to the World Health Organization (WHO). Outbreak investigation which is based on a critical assessment of core components of infection prevention and control (IPC) programs allows for the identification of different sources of exposure to the COVID-19 virus and for informing additional IPC recommendations. To date, the Democratic Republic of the Congo (DRC) is categorized as a high-risk country due to weaknesses in the health system, low capacity for diagnosis, socioeconomic characteristics of the population, and insufficient vaccination coverage.

**Aim:**

To investigate the burden of COVID-19 among HCWs and identification of IPC gaps to reduce HCWs-associated infection at different levels (facilities, communities, and points of entry) following the WHO strategy for IPC program implementation during the first to the third wave of the pandemic.

**Methods:**

A retrospective cohort study was conducted using the DRC National Department of Health (NDOH) database and WHO questionnaire suspected and confirmed COVID-19 cases among HCWs from 10/03/2020 to 22/06/2021. The investigation was conducted by a trained IPC response team to identify the sources of the exposures. The questionnaire included demographics, profession, types of interaction between HCWs and patients, and community-based questions regarding family members and other behaviors. These variables were assessed using a multimodal strategy framework. Knowledge and adherence to IPC gaps using WHO guidelines were performed for each COVID-19-positive or suspected HCW. WHO rapid Scorecard dashboard was conducted for evaluating healthcare facilities (HCFs) performance during the COVID-19 pandemic.

**Results:**

Cumulative incidence of positive HCWs was 809 /35,898(2.2%) from the first to the third wave of COVID-19 among 6 provinces of DRC. The distribution of the HCWs infected by COVID-19 was predominated by nurses (42%), doctors (27%), biologists (8%), environmental health practitioners (5%), interns (3%), and other categories (15%). Other categories included nutritionists, physiotherapists, midwives, pharmacists, and paramedics. The investigation revealed that about 32% of HCWs were infected from household contacts, 11% were infected by HCFs, 35% were infected in the community and 22% were infected from unknown exposures. The mean score of IPC performance for all evaluated HCFs was 27/42(64%). This shows that IPC performance was moderate. Lower or minimal performance was noted in the implementation of the IPC program at the national and facility level, triage and screening, isolation handwashing and multimodal strategies of hand hygiene, PPE availability, and rationale, waste segregation, waste disposal, sterilization, and training of HCWs.

**Conclusion:**

This study revealed that the prevalence of HCWs who tested positive for the COVID-19 virus was high among frontline healthcare workers from 6 provinces of DRC. A high prevalence of nosocomial infection was correlated with insufficient IPC adherence in the context of COVID-19. Strategies to strengthen IPC capacity building and provide HCWs with sufficient PPE stocks and budgets may improve IPC performance in the Democratic Republic of the Congo. This will further allow for adherence to WHO recommendations for successful program implementation to minimize COVID-19 transmission in HCFs, communities, and public gatherings. And this may be transferable to other infectious diseases.

## Background

Health-care workers (HCWs) are at the frontline of response of coronavirus disease 2019 (COVID-19) pandemic [1–3]. Protecting HCWs is of paramount importance to World Health Organization (WHO) as it ensures continuity of patient care in health care systems [4]. This makes investigating the burden of COVID-19 in HCWs imperative. Outbreak investigation which is based on critical assessment of core components of infection prevention and control (IPC) programs allows for the identification of different sources of exposures to COVID 19 and for informing additional IPC recommendations [4, 5]. Knowledge of IPC procedures among HCWs is crucial for reducing the likelihood of occupational risk and is pivotal in reinforcing compliance with IPC measures at the healthcare facility level, public places and communities. A systematic review and meta-analysis investigated the incidence and risk factors associated with COVID-19 among HCWs using reverse transcription-polymerase chain reaction (RT-PCR) and showed an infection rate 11% (95% confidence interval (CI): 7, 15) in 2020 (Ref). Among HCWs who tested positive for COVID-19 using RT-PCR, 40% (95% CI: 17, 65) were asymptomatic at time of diagnosis. Finally, severe clinical complications developed in 5% (95% CI: 3, 8) of the COVID-19-positive HCWs, and 0.5% (95% CI: 0.02, 1.3) died [3]. HCWs are at risk for acquiring COVID-19 by working in close proximity to patients and coworkers, and insufficient adherence to IPC measures may increase this risk. One study reported that HCWs accounted for 3.8 to 19% of COVID-19 cases before COVID-19 vaccination rollout out program. Strategies to reduce the risk of acquiring COVID-19 in the health care setting include vaccination, adherence on IPC measures and strong surveillance system [3]. The goals of IPC program during the outbreaks at facility levels including reduction of transmission of health-care associated infections and thereby to enhance the safety of all who are present in a health-care facility, patients, staff and visitors. To enhance the ability of a health-care facility to respond to an outbreak. To lower or eliminate the risk of the health-care facility itself amplifying the outbreak [3, 6–8]. To date, the DRC is categorized high risk country due to a weak health care system, low capacity for diagnosis, poor socioecomic characteristics of the population and insufficient vaccination coverage. Regarding the low vaccination coverage, low vaccine availability, misinformation, and insufficient campaigns to debunk coronavirus myths have played a role. Regarding the low vaccination coverage, low vaccine availability, misinformation, and insufficient campaigns to debunk coronavirus myths have played a role. Currently, the Democratic Republic of the Congo has administered at least **3,825,485** doses of COVID vaccines so far, which is only about **2.2%** of the country’s population [3, 6].

It is important to note that the DRC has seen a rise in infectious diseases including measles, and Ebola over the past years [3]. Similar to other countries worldwide, the burden introduced by COVID-19 to the healthcare system has significantly set back the epidemiological control of other infectious diseases. Documenting the impact of COVID-19 on HCWs is therefore not only important for COVID-19 but for future outbreaks and pandemics that may occur. This study was conducted to investigate the burden of COVID-19 among HCWs and identification of IPC gaps which contribute to HCWs-associated infection at different levels (facilities, communities and point of entries) following the WHO strategy for IPC program implementation from the first to the third wave of the outbreak [6–9]. Various studies have identified the similar gaps for both EVD and Ebola transmission among HCW due to insufficient of IPC practices at service delivery [6–14]. .

## Methods and materials

### Design and setting

A retrospective cohort study was conducted using the DRC National Department of Health (NDOH) database and a WHO questionnaire was conducted for each suspected and confirmed COVID-19 cases among HCW from 10/03/2020 to 22/06/2021 [4]. Study was conducted based in light of Declaration of Helenski for protection of human details and right of informed consents prior recruitment into the study [15].

The DRC is a large country with an estimated population of 90 million. The population of the DRC is relatively young: 62.7% are between 0 and 24 years, 30.9% between 25 and 54 years, and 6.3% ≥55 years of age. The average age is. The country is composed of 26 provinces and shared borders with 9 countries. In terms of the operational health system structure, the DRC has 516 health zones [16].

The investigation was conducted by trained IPC response team to identify the sources of the exposures. The questionnaire included demographic, profession, types of interaction between HCW and patients, community-based questions regarding family members, and other behaviors were assessed using a multimodal strategy framework. Knowledge and adherence on IPC gaps using WHO guidelines were performed for each positive or suspected HCWs. A WHO rapid Scorecard dashboard was conducted for evaluation of healthcare facilities (HCF) performance during COVID-19 pandemic. Approximately 935 HCFs were assessed using WHO electronic Scorecard dashboard. 247 HCFs were categorized as primary care, 691 and 22 were from secondary and tertiary settings respectively. From these, 19 exclusive COVID-19 clinics, 157 were mixed HCFs and 776 were non COVID-19 related facilities. These facilities were assessed from 72 Districts of DRC, which comprised 317 health areas. The questionnaire included 42 questions which divided into 14 major components of IPC. Data was described in Excel spreadsheet and analysed in STATA version 16.

The study was approved by the DRC National Ethics Committee. Study was conducted based in light of Declaration of Helenski for protection of human details and right of informed consents prior recruitment into the study [15]. The samples collected in this by the scientists working during this periods confirmed that all experiments were performed in accordance with relevant guidelines and regulations. All methods were carried out in accordance with relevant guidelines provided and policies [15]. The written consents consents were obtained from all subjects and/or their legal guardian(s). The study included the adults from 18 years not children’s or minors [15].

## Results

Six provinces participated in the study. From these, the incidence of infected HCWs were 9, 9% in Kongo Central, 5% in Ituri, 4.5% in Nork Kivu, 4.9% in South Kivu, 1.4% in Kinshasa and 1.0% in Haut Katanga (Table 1).

Cumulative incidence of positive HCWs was 809/35,898 (2.2%) from first to third wave of COVID-19 among 6 provinces of DRC as shown in Table 1.

**Table 1:** Summary of HCWs COVID-19 infections in 6 provinces from the DRC.

Province	COVID-19 positive overall (n)	HCWs tested positive (n)	Incidence of COVID-19 positive HCWs
**Kinshasa**	**27,550**	**397**	**1.4%**
**Kongo Central**	**1931**	**192**	**9.9%**
**Nord Kivu**	**3048**	**136**	**4.5%**
**Sud Kivu**	**947**	**46**	**4.9%**
**Ituri**	**339**	**17**	**5.0%**
**Haut Katanga**	**2083**	**21**	**1.0%**
**TOTAL**	**35,898**	**809**	**2.2%**

**Fig. 1:**
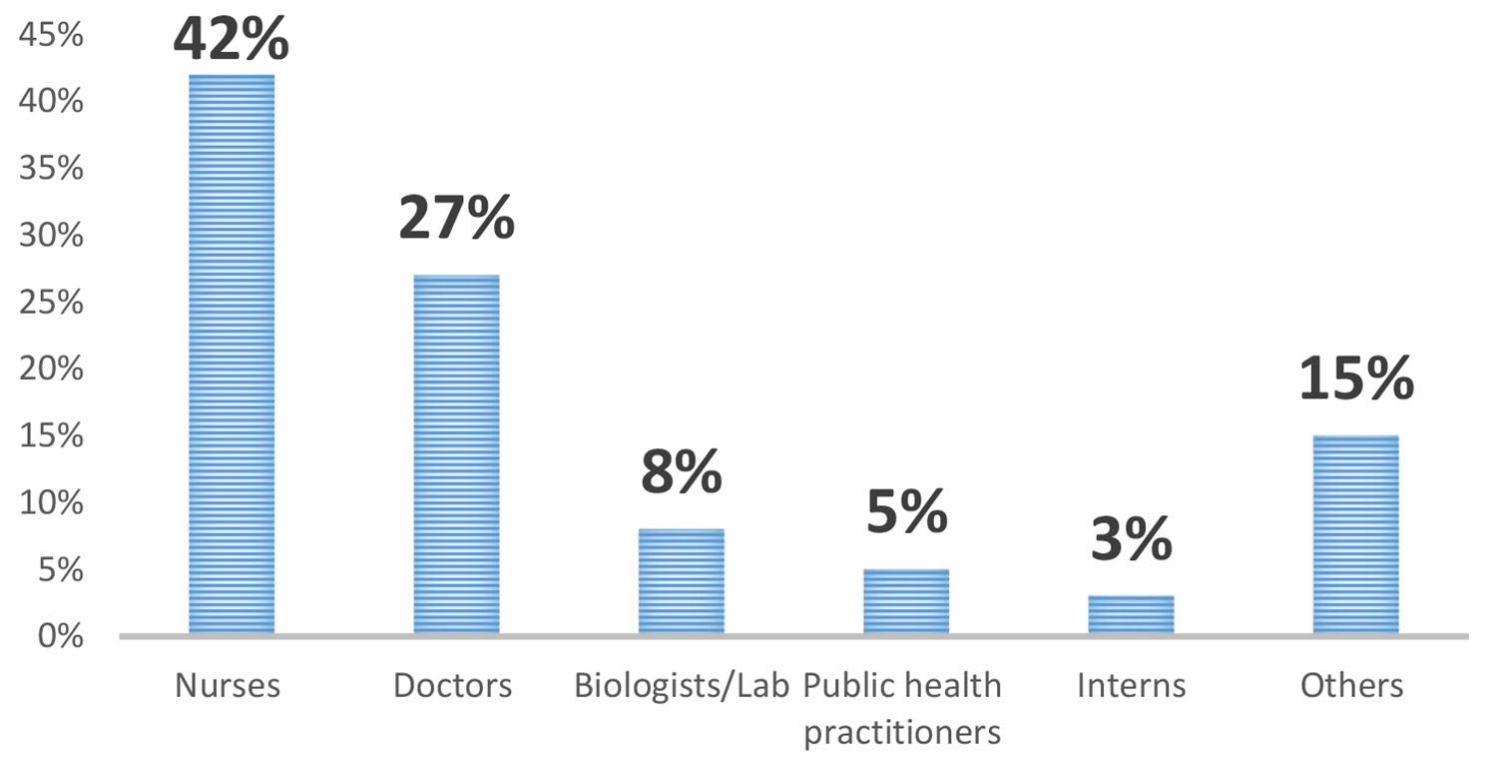
Distribution of COVID-19 positive HCWs by job category

The distribution of the HCWs infected from COVID-19 was predominated by nurses (42%), followed by doctors (27%), biologists (8%), environmental health practitioners (5%), interns (3%), and other categories (15%). Other categories included nutritionists, physiotherapists, midwives, pharmacists and paramedics. Figure 1 provides the distributions of HCWs tested COVID-19 positive according to job category.

**Fig. 2:**
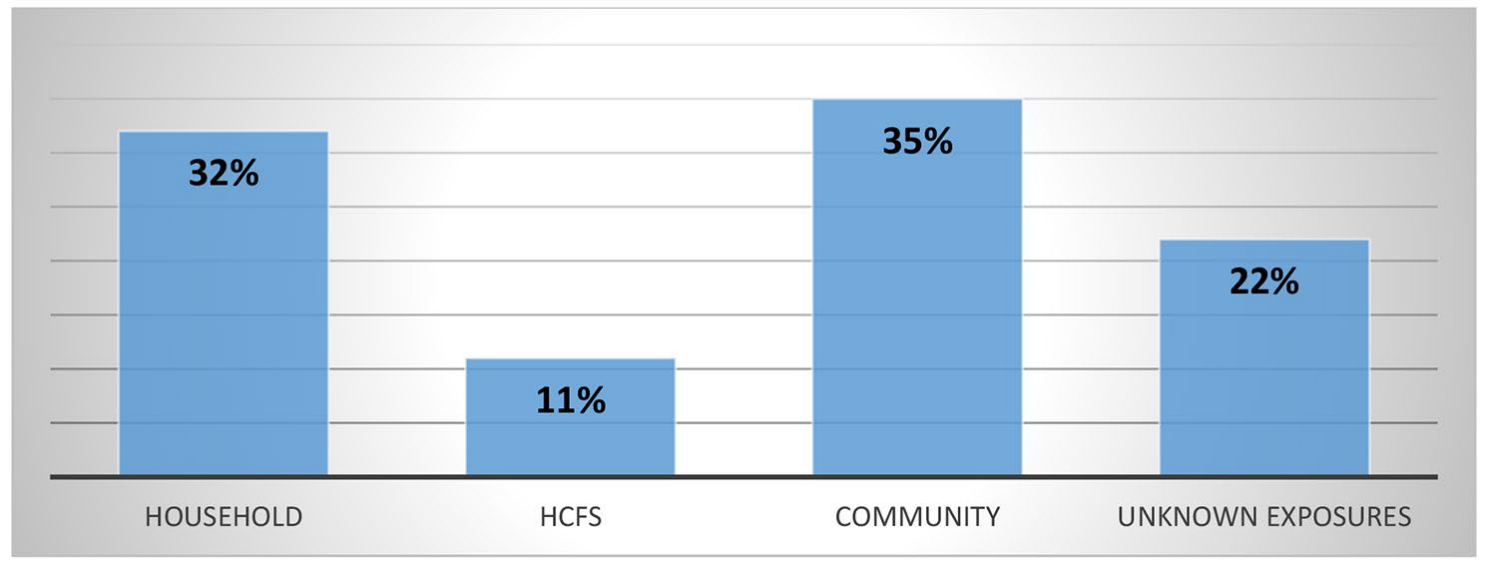
Sources and places of exposure among COVID-19 positive HCWs

The investigation revealed that about 32% of HCWs were infected from household contacts, 11% HCWs were infected from HCFs, 35% were infected in the community and 22% were infected from unknown exposures (Fig. 2).

**Fig. 3:**
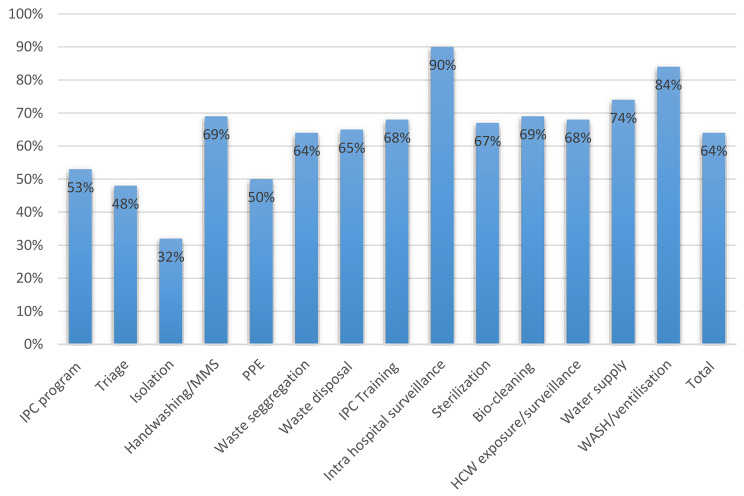
IPC performance of HCFs assessed from 6 provinces of DRC using the WHO rapid Scorecard dashboard

The mean score IPC performance for all evaluated HCFs using the WHO rapid Scorecard dashboard was 27/42 (64%), showing moderate IPC performance. Low or minimal performance ($\le$50) was identified in triage and screening, isolation and PPE availability. Moderate performance (>50-70%) was noted in implementation of the IPC program at national and facility level, isolation handwashing and multimodal strategies of hand hygiene, waste segregation, waste disposal, sterilization and training of HCWs. IPC performance was sufficient for intra hospital…(90%), and WASH/ventilation (84%). Figure 3 provides the summary of IPC performances from different provinces.

## Discussion

This study provides epidemiological roadmap of HCW COVID-19 infection among frontline HCW in 6 provinces of DRC with the purpose of implementing policy guidelines for protection of HCWs working in low resource settings in a pandemic. WHO tools for outbreak investigation among HCW were utilized for the identification of sources of exposures for COVID-19 positive HCWs [5]. IPC scorecard revealed that insufficient IPC trainings, lack of IPC focal points in many facilities, insufficient storage of PPE, insufficient IPC program implementation at facility and lack of dedicated budgets for IPC practitioners may play important role in the overall IPC performance in DRC and contribute to HCWs infection rates. There is an overall scarcity of occupational health physicians who can conduct in-depth investigations on sources of exposures for COVID-19 infected HCWs. The consequences of this scarcity is that sources of exposure for 22% of HCWs were not known which could facilitate additional nosocomial transmissions or community based transmission. More efforts are needed to support and strengthen IPC program implementation as well as development of country policy guidelines for protection of HCWs in terms of compensation, cascade training and appointment of certified IPC focal points in each HCF to control COVID-19 pandemic and other communicable diseases such as HIV, TB, typhoid fever, EVD, yellow fever and antimicrobial resistance.

This preliminary descriptive analysis is correlated with different studies reported globally [7]. Nurses are likely to be affected by COVID-19 due to proximity with suspected or positive cases in the healthcare setting without well implemented IPC multimodal strategy. Similar to our study doctors and clinical associates were the second high risk category, after nurses [7]. The global WHO IPC survey conducted in South Africa identified several gaps associated with IPC program implementation from national to facility level. These included scarcity of IPC appointed focal persons, disjunction between the national and provincial levels, insufficient monitoring and evaluation activities reporting and minimal knowledge on HCW-associated infection investigation and management and policy issues between different IPC stakeholders such as occupational health and safety, environmental health, waste management and quality assurance [8]. Although currently, COVID-19 cases in DRC have significantly declined, like in the rest of the world, comprehensive safety measures are being implemented locally to protect HCWs and patients. A study conducted in 23 referral hospitals located in three towns of the DRC (Lubumbashi, Kamina, Mbuji-Mayi) showed that most Congolese HCWs had sufficient knowledge on COVID-19, whereas the majority did not comply with consistent PPE use [9]. Whereas, a meta-analysis conducted to determine the seroprevalence of SARS-CoV-2 antibodies among HCWs estimated overall seroprevalence among HCWs was 8.7% (95% confidence interval 6.7-10.9%) Seroprevalence was higher in studies conducted in North America (12.7%) compared with those conducted in Europe (8.5%), Africa (8.2) and Asia (4%). Meta-regression showed that increased sensitivity of antibody tests was associated with increased seroprevalence. Some factors were associated with the increased risk of transmission. These included male gender, Black population, Asian and Hispanic HCWs, working in COVID-19 wards, patient-related works, front-line HCWs, healthcare assistants, shortage of PPE, self-reported belief of previous SARS-CoV-2 infection, previous positive RT-PCR test; and household contact with suspected or confirmed cases of COVID-19 [6].

On 3 June 2021, the International Council of Nurses (ICN) implored governments to compile data on HCW withCovid-19 infections and deaths. Currently, there is no standardized global registry to estimate the incidence of COVID-19 among HCWs, although it has infected an estimated 230 000 and led to the deaths of 600 nurses. Analysis by the ICN suggests that on average, 7% of all Covid-19 cases worldwide occur among HCWs. Notable exceptions include some high-income countries (USA, Spain and Ireland) where HCWs account for 15–30% of all infections [10]. Other authors have reported the challenges of IPC practice and implementation gaps in many countries, including Africa [11–14].

Lessons learnt from South Africa revealed that the South Africa Department of Health developed COVID-19 IPC guidelines, engaged relevant stakeholders and these guidelines were published and disseminated throughout the provinces [10]. IPC trainings were initiated and aligned to the facility’s needs. IPC trainings were coordinated and supported by NDOH, academic institutions, WHO, Africa CDC and ICAN. National IPC strategic Framework as well as manual for implementation were approved and issued for implementation to Provincial IPC stakeholders. Quality assurance managers are responsible for IPC program in South Africa. As of 12 August 2020, a cumulative total of 568 919 confirmed COVID-19 cases in South Africa were recorded [10]. About 27 360 HCW were reported among positive cases (5% which is below the global average of 10%) Of those, 6027 (22%) were from the private sector and 21 333 (78%) were from the public sector. Among these, 1 644 (6%) of these HCWs were doctors, 14 143 (52%) were nurses, 28 (less than 1%) were Port Health workers and 11 545 (42%) were from other categories of health workers [10]. A study done in India showed a similar prevalence of 5% of frontline HCWs (hospital-acquired COVID-19) and the authors cited several factors including the depletion of the facility’s workforce, increasing workload, as well as exposure to patients outside quarantine, as contributing to this infection rate. The study highlighted the need for “advance national programs for HCW safety, and connecting HCW safety policies to existing patient safety policies” which are part of WHO recommendations for protecting HCWs [11]. Addressing issues of mental health for HCWs especially during pandemics and disasters in important [3, 11].

### Limitations and recommendations

Data on HCW safety during the COVID-19 pandemic is a high priority as it is underreported, especially data on the comorbidities, risk factors, source of exposures, socioeconomic characteristics, race and disparities between patient-facing and non-patient-facing front-line HCWs. Some reasons for insufficient investigation of HCW infections maybe due lack of occupational specialists in many African countries, policy and ethics concerns regarding HCW data reporting and insufficient knowledge on HCW risk of exposure management and investigation. In general, IPC is considered to be a new program in Africa with insufficient IPC practitioners without well-defined academic programs and lack of financing for program implementation. Therefore, establishment of a permanent structured IPC team at all levels should facilitate program implementation and reduction in nosocomial infections. IPC plans and organograms at the national, provincial and Districts to be developed, discussed, finalised and approved between stakeholders in AFRO/Region [10–14].

## Conclusion

This study revealed that the prevalence of HCWs who tested positive from COVID-19 virus was high among frontline healthcare workers from 6 provinces of DRC. Insufficient IPC adherence may have played a role increasing the number of COVID-19 nosocomial infections in the DRC. Strategies to strengthen IPC capacity building and providing HCWs with sufficient PPE stocks and budgets may improve IPC performance in this country to comply with WHO recommendations for successful program implementation to minimize COVID-19 transmission in HCFs, communities and public gatherings.

## Data Availability

The datasets supporting the conclusions of this article are available through the AHRI data repository (District Health Information System 2.0), known as DHIS. Data can be requested directly from this repository and the corresponding author (DRC department of health and WHO/AFRO surveillance manager following the ethic approval of the country Minister of Health.

## Abbreviations

HAI: healthcare associated infections
IPC: infection Prevention and control
HCF: Healthcare facilities
HCW: Healthcare workers
